# Supplementary material for: Early language exposure affects neural mechanisms of semantic representations
Source: eLife. 2023 May 10;12:e81681. doi: 10.7554/eLife.81681 (PMC10238089; doi:10.7554/eLife.81681)
Supplement: Figure 1—source data 1. [file elife-81681-fig1-data1.docx]

**Figure 1-Source data 1**

Ninety words in the fMRI task, grouped into ten semantic clusters based on k-means clustering of the group-mean hearing semantic space.

| No. | Cluster labels | Words |
| --- | --- | --- |
| Cluster 1 | Animals | 熊猫 (panda), 老鼠 (rat), 老虎 (tiger), 大象 (elephant), 麻雀 (sparrow), 乌龟 (tortoise), 蚂蚁 (ant), 兔子 (rabbit), 猫 (cat), 长颈鹿 (giraffe) |
| Cluster 2 | Face/Body parts | 肩膀 (shoulder), 胳膊 (arm), 大腿 (thigh), 鼻子 (nose), 眼睛 (eye), 嘴唇 (lips), 耳朵 (ear), 膝盖 (knee), 手指 (finger), 脚踝 (ankle) |
| Cluster 3 | Large objects | 空调 (air conditioner), 冰箱 (refrigerator), 电视 (television), 微波炉 (microwave), 洗衣机 (washing machine), 柜子 (cabinet), 椅子 (chair), 沙发 (sofa), 桌子 (table), 床 (bed) |
| Cluster 4 | Tools | 勺子 (spoon), 剪刀 (scissors), 斧头 (axe), 鼠标 (computer mouse), 筷子 (chopsticks), 扫帚 (broom), 牙刷 (toothbrush), 钥匙 (key), 铅笔 (pencil), 锤子 (hammer) |
| Cluster 5 | Positive emotions | 兴奋 (excited), 舒心 (comfortable), 快乐 (happy), 骄傲 (proud) |
| Cluster 6 | Negative emotions | 敌意 (hostility), 愤怒 (anger), 恐惧 (fear), 冷漠 (apathy), 难过 (sad), 反感 (antipathy), 沮丧 (depressed) |
| Cluster 7 | Positive abstract words | 爱心 (loving/caring), 慈善 (charity), 魔力 (magic), 奇迹 (miracle), 风景 (scenery), 天堂 (heaven), 光彩 (splendor) |
| Cluster 8 | Negative abstract words | 暴力 (violence), 骗局 (fraud), 纠纷 (dispute), 债务 (debt), 创伤 (trauma), 疾病 (disease), 死亡 (death), 过失 (fault), 错误 (error) |
| Cluster 9 | Social words | 缘分 (fate), 友情 (friendship), 婚姻 (marriage), 制度 (system), 社会 (society), 关系 (relationship), 地位 (status), 身份 (identity), 义务 (obligation), 买卖 (business), 协议 (agreement), 团队 (team), 纪律 (discipline) |
| Cluster 10 | Low-emotional, low-social | 概念 (concept), 内容 (content), 性质 (characteristic), 数据 (data), 作用 (effect), 结果 (result), 原因 (reason), 过程 (process), 方法 (method), 现象 (phenomenon) |
