# Supplementary material for: Early language exposure affects neural mechanisms of semantic representations
Source: eLife. 2023 May 10;12:e81681. doi: 10.7554/eLife.81681 (PMC10238089; doi:10.7554/eLife.81681)
Supplement: Figure 1—source data 3. [file elife-81681-fig1-data3.docx]

**Figure 1-Source data 3**

Details of semantic ROIs, including cluster location, extent, peak t values, and MNI coordinates.

| Cluster name | Cluster extent | Peak *t* value | Maxima MNI coordinates | | |
| --- | --- | --- | --- | --- | --- |
|  | (voxels) |  | x | y | z |
| **L TPOsup, IFGtriang, MTG, STG, ORBinf, TPOmid, ITG** | **1297** | **6.98** | **-48** | **16** | **-18** |
|  |  | **5.88** | **-54** | **0** | **-16** |
|  |  | **5.85** | **-52** | **24** | **6** |
| R TPOsup, MTG, TPOmid, STG | 746 | 6.30 | 58 | 10 | -16 |
|  |  | 5.83 | 56 | 0 | -18 |
|  |  | 5.64 | 42 | 10 | -20 |
| **L MTG** | **516** | **6.09** | **-54** | **-28** | **0** |
|  |  | **5.13** | **-52** | **-42** | **0** |
|  |  | **4.95** | **-60** | **-46** | **0** |
| L SFGmed | 115 | 5.06 | -6 | 60 | 28 |
|  |  | 4.07 | -8 | 60 | 20 |
| B PCG, R PCUN | 119 | 4.89 | 2 | -46 | 24 |
|  |  | 4.34 | 6 | -42 | 16 |
| R STG, MTG | 109 | 4.68 | 50 | -26 | 0 |
|  |  | 4.15 | 58 | -28 | 0 |
|  |  | 3.83 | 44 | -38 | 2 |

Notes: These regions were functionally localized as those showing stronger activations to abstract/nonobject words than concrete/object words in 33 hearing participants and surviving the the threshold of voxel-level *p* < .001, cluster-level FWE-corrected *p* < .05. Regions in bold fonts were considered as semantic ROIs for subsequent RSA and univariate abstractness analyses. For each region, two or three local maxima more than 8.0 mm apart are shown. L, left hemisphere; R, right hemisphere; B, bilateral. IFGtriang, inferior frontal gyrus, triangular part; ITG, inferior temporal gyrus; MTG, middle temporal gyrus; ORBinf, inferior frontal gyrus, orbital part; PCG, posterior cingulate gyrus; PCUN, precuneus; SFGmed, superior frontal gyrus, medial; STG, superior temporal gyrus; TPOmid, temporal pole: middle temporal gyrus; TPOsup, temporal pole: superior temporal gyrus.
