# Supplementary material for: Early language exposure affects neural mechanisms of semantic representations
Source: eLife. 2023 May 10;12:e81681. doi: 10.7554/eLife.81681 (PMC10238089; doi:10.7554/eLife.81681)
Supplement: Figure 2—source data 2. [file elife-81681-fig2-data2.docx]

**Figure 2-Source data 2**

Cluster details of the whole-brain searchlight RSA results.

| Cluster name | Cluster extent  (voxels) | Cluster-level p (FWE corrected) | Peak *t* value | Maxima MNI coordinates | | |
| --- | --- | --- | --- | --- | --- | --- |
|  |  |  |  | x | y | z |
| *Native > delayed (df = 37)* |  |  |  |  |  |  |
| L MTG, TPOsup | 162 | 0.22 | 5.87 | -60 | 6 | -20 |
| L SFGmed, SFGdor | 114 | 0.38 | 4.35 | -8 | 58 | 14 |
| L THA | 47 | 0.78 | 4.07 | -16 | -10 | 4 |
| L SFGmed | 30 | 0.89 | 4.01 | -8 | 40 | 42 |
| L ANG, MOG | 48 | 0.78 | 3.98 | -48 | -72 | 36 |
| B ORBsupmed, ACG | 101 | 0.45 | 3.96 | -12 | 50 | -6 |
|  |  |  | 3.81 | -2 | 54 | -8 |
| L MTG | 48 | 0.78 | 3.91 | -52 | -24 | -14 |
| L SFGmed, DCG | 38 | 0.84 | 3.85 | -8 | 26 | 36 |
| R HIP | 16 | 0.96 | 3.64 | 22 | -12 | -14 |
| *Delayed > native (df = 37)* |  |  |  |  |  |  |
| L PHG | 15 | 0.96 | 4.13 | -20 | -10 | -32 |
| *Native signers (n = 16)* |  |  |  |  |  |  |
| L MTG, STG, TPOsup, ITG | 442 | 0.004 | 7.45 | -58 | 4 | -18 |
|  |  |  | 4.86 | -50 | -22 | -14 |
| L ORBinf, ORBmid | 395 | 0.006 | 7.35 | -30 | 32 | -14 |
|  |  |  | 5.45 | -24 | 46 | -18 |
| L MTG, ANG, MOG, SMG, IPL, ITG, STG | 2068 | <0.001 | 6.95 | -50 | -52 | 22 |
|  |  |  | 6.66 | -48 | -42 | 32 |
|  |  |  | 6.33 | -48 | -72 | 36 |
| L SFGdor, B SFGmed | 730 | <0.001 | 6.95 | -10 | 52 | 34 |
|  |  |  | 6.15 | -10 | 58 | 26 |
|  |  |  | 5.92 | -12 | 60 | 18 |
| L IFGtriang, MFG | 492 | 0.003 | 5.72 | -40 | 32 | 26 |
|  |  |  | 5.08 | -48 | 32 | 20 |
|  |  |  | 5.03 | -50 | 28 | 12 |
| *Delayed signers (n = 23)* |  |  |  |  |  |  |
| L PHG | 54 | 0.72 | 5.76 | -20 | -8 | -30 |
| R ANG, IPL | 145 | 0.22 | 5.72 | 40 | -60 | 40 |
| L MOG, SOG | 195 | 0.11 | 5.18 | -28 | -72 | 34 |
|  |  |  | 3.82 | -38 | -74 | 28 |
| L IPL | 186 | 0.13 | 4.94 | -52 | -40 | 48 |
|  |  |  | 3.83 | -42 | -38 | 38 |
| L TPOsup | 25 | 0.92 | 4.84 | -46 | 12 | -24 |
| R SFGdor | 13 | 0.98 | 4.25 | 18 | 54 | 16 |
| L ORBmid, ORBsup, ORBinf | 37 | 0.85 | 4.25 | -22 | 38 | -12 |
| L IFGtriang | 52 | 0.74 | 4.18 | -44 | 34 | 16 |
| L MOG | 24 | 0.93 | 4.16 | -34 | -90 | 18 |
| L FFG, ITG | 24 | 0.93 | 4.12 | -38 | -16 | -30 |
| L PoCG, IPL | 12 | 0.98 | 3.93 | -28 | -42 | 48 |
| L CAU | 12 | 0.98 | 3.88 | -6 | 10 | -4 |
| R ORBsup, ORBinf | 21 | 0.94 | 3.85 | 24 | 32 | -12 |
| L ITG | 26 | 0.92 | 3.79 | -56 | -52 | -12 |
|  |  |  | 3.78 | -50 | -50 | -4 |

Notes: Clusters were reported at the threshold of voxel-level *p* < .001, cluster size > 10 voxels, except for the results of native signers, which were thresholded at voxel-level *p* < .001, cluster-level FWE-corrected *p* < .05. Two or three local maxima more than 8.0 mm apart were shown. L, left hemisphere; B, bilateral; R, right hemisphere; ACG, anterior cingulate and paracingulate gyrus; ANG, angular gyrus; CAU, caudate; DCG, median cingulate and paracingulate gyrus; HIP, hippocampus; IFGtriang, inferior frontal gyrus, triangular part; IPL, inferior parietal lobule; ITG, inferior temporal gyrus; MFG, middle frontal gyrus; MOG, middle occipital gyrus; MTG, middle temporal gyrus; ORBinf, inferior frontal gyrus, orbital part; ORBmid, middle frontal gyrus, orbital part; ORBsupmed, superior frontal gyrus, medial orbital; PHG, parahippocampus; SFGdor, superior frontal gyrus, dorsolateral; PoCG, postcentral gyrus; SFGmed, superior frontal gyrus, medial orbital; SMG, supramarginal gyrus; SOG, superior occipital gyrus; STG, superior temporal gyrus; THA, thalamus; TPOsup, temporal pole: superior temporal gyrus.
